# Two Modes of Regulation of the Fatty Acid Elongase ELOVL6 by the 3-Ketoacyl-CoA Reductase KAR in the Fatty Acid Elongation Cycle

**DOI:** 10.1371/journal.pone.0101823

**Published:** 2014-07-08

**Authors:** Tatsuro Naganuma, Akio Kihara

**Affiliations:** Laboratory of Biochemistry, Faculty of Pharmaceutical Sciences, Hokkaido University, Sapporo, Japan; Indian Institute of science, India

## Abstract

Fatty acids (FAs) are diverse molecules, and such diversity is important for lipids to exert their functions under several environmental conditions. FA elongation occurs at the endoplasmic reticulum and produces a variety of FA species; the FA elongation cycle consists of four distinct enzyme reactions. For this cycle to be driven efficiently, there must exist coordinated regulation of protein components of the FA elongation machinery. However, such regulation is poorly understood. In the present study, we performed biochemical analyses using the FA elongase ELOVL6 and the 3-ketoacyl-CoA reductase KAR, which catalyze the first and second steps of the FA elongation cycle, respectively. *In vitro* FA elongation assays using membrane fractions demonstrated that ELOVL6 activity was enhanced ∼10-fold in the presence of NADPH, although ELOVL6 itself did not require NADPH for its catalysis. On the other hand, KAR does use NADPH as a reductant in its enzyme reaction. Activity of purified ELOVL6 was enhanced by ∼3-fold in the presence of KAR. This effect was KAR enzyme activity-independent, since it was observed in the absence of NADPH and in the KAR mutant. However, ELOVL6 enzyme activity was further enhanced in a KAR enzyme activity-dependent manner. Therefore, KAR regulates ELOVL6 via two modes. In the first mode, KAR may induce conformational changes in ELOVL6 to become structure that can undergo catalysis. In the second mode, conversion of 3-ketoacyl-CoA to 3-hydroxyacyl-CoA by KAR may facilitate release of the product from the presumed ELOVL6–KAR complex.

## Introduction

Fatty acids (FAs), which are components of most cellular lipids, are diverse molecules in terms of chain length and degree of unsaturation. FA diversity arises when palmitic acid (C16∶0) produced by FA synthase (FAS) or FAs taken from foods are converted to other FA species by elongation and/or desaturation. Such diversity in FAs enables lipid molecules to fulfill their organism-, tissue-, cell-, and organelle-specific functions and to respond to changes of environment. Most cellular FAs are long-chain FAs (C11 to C20), among which C16 and C18 FAs are the most abundant [1]. However, very long-chain FAs (VLCFAs) with >C20 also exist and play several important functions in which long-chain FAs cannot be substituted [2], [3]. Saturated and monounsaturated VLCFAs are specifically used for sphingolipid production [2], [3], [4], whereas most polyunsaturated VLCFAs are incorporated into glycerophospholipids [5], [6], [7].

**Figure 1:**
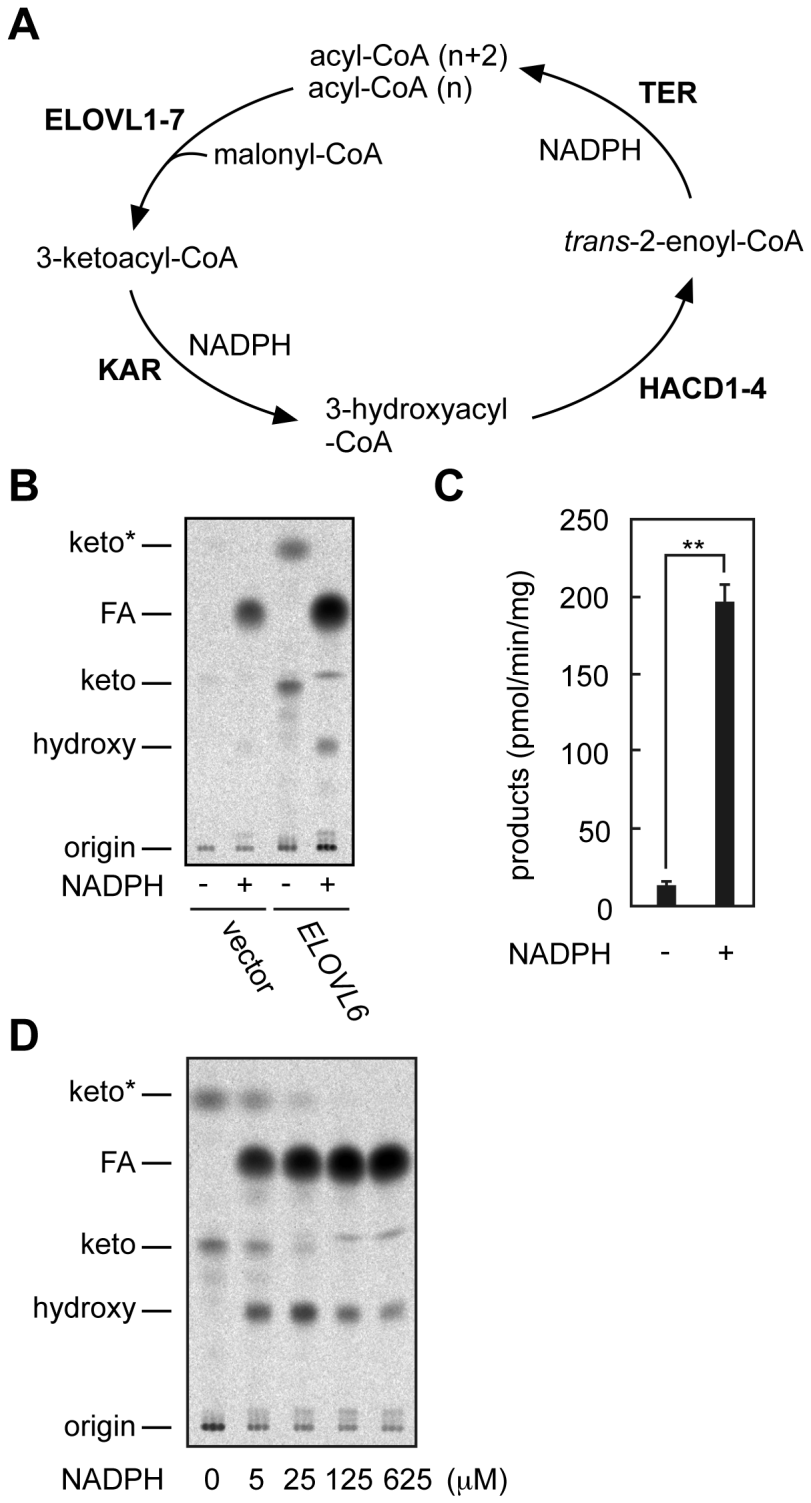
ELOVL6 activity in membrane fractions is enhanced in the presence of NADPH. (A) The FA elongation cycle consisting of the reaction steps of condensation, reduction, dehydration, and reduction and the mammalian enzymes involved in each step are illustrated. (B-D) HEK 293T cells were transfected with pCE-puro 3xFLAG-1 (vector; B) or pCE-puro 3xFLAG-ELOVL6 (B-D). Total membrane fractions (10 µg) prepared from them were incubated with 8 µM C16∶0-CoA and 27.3 µM [^14^C]malonyl-CoA (1.5 µCi/ml) for 30 min at 37°C in the presence or absence of 1 mM NADPH (B and C) or in the presence of NADPH at indicated concentrations (D). After termination of the reactions, lipids were saponified, acidified, extracted, and separated by normal-phase TLC followed by detection (B and D) and quantification (C) with a bioimaging analyzer BAS-2500 (Fuji Photo Film, Tokyo, Japan). Values presented in (C) are the sum of the products in (B) obtained from ELOVL6-overproducing cells and represent the mean ± S.D. from three independent experiments. Statistically significant differences are indicated (**p<0.01; *t*-test). keto, 3-keto-FA; *keto, a by-product of 3-ketoacyl-CoA, perhaps a decarboxylated form, generated during the saponification reaction; hydroxy, 3-hydroxy-FA.

FAs are elongated in forms of acyl-CoAs through the FA elongation cycle by the endoplasmic reticulum-resident FA elongation machinery [2], [8]. The FA elongation cycle includes four reactions: condensation, reduction, dehydration, and reduction (Fig. 1A). In the first, rate-limiting step, acyl-CoA is condensed with malonyl-CoA to form 3-ketoacyl-CoA. In mammals, this step is catalyzed by one of the seven FA elongases (ELOVL1–7). Each FA elongase exhibits characteristic substrate specificity towards acyl-CoA substrate [9]. The second step is catalyzed by 3-ketoacyl-CoA reductase (KAR), which reduces 3-ketoacyl-CoA to 3-hydroxyacyl-CoA using NADPH as a cofactor [10]. Then, 3-hydroxyacyl-CoA is dehydrated to *trans*-2-enoyl-CoA by one of the four 3-hydroxyacyl-CoA dehydratases (HACD1-4) [11]. Finally, the *trans*-2-enoyl-CoA reductase, TER, catalyzes the conversion of *trans*-2-enoyl-CoA to an acyl-CoA with two more carbon units than the original acyl-CoA in an NADPH-dependent manner [10].

Evidence demonstrating the pathological and physiological importance of FA diversity is increasing from analyses of human disorders and of mice with knockouts (KOs) of the genes encoding the components of the FA elongase machinery. The FA elongase ELOVL6 is responsible for the conversion of C16-CoAs to C18-CoAs [9], [12], [13]. In *Elovl6* KO mice, ≥C18 FAs are decreased, whereas ≤C16 FAs are increased. This change in FA composition protects the KO mice against high fat diet-induced insulin resistance [14], but causes pulmonary fibrosis [15]. ELOVL1 and ELOVL4 are responsible for the production of saturated and monounsaturated VLCFAs with lengths of C22-C26 and ≥C28, respectively [9], [16]. Epidermal ceramides, the hydrophobic backbones of sphingolipids, specifically contain saturated and monounsaturated ≥C26 FAs and are important for skin barrier function [17]. Both *Elovl1* and *Elovl4* KO mice are neonatal lethal due to skin barrier defects [18], [19], [20]. Moreover, mutations in the human *ELOVL4* gene have been found [21], [22]. Recessive mutations cause the cutaneous disorder of ichthyosis as well as neural symptoms such as intellectual disability and spastic quadriplegia [22]. On the other hand, dominant mutations cause a kind of juvenile macular dystrophy (Stargardt disease 3) due to a decrease in retinal phosphatidylcholines with C32–C36 polyunsaturated FAs [21], [23]. ELOVL2 and ELOVL5 catalyze the elongation of polyunsaturated acyl-CoAs with C20–C22 and C18–C20, respectively [9]. *Elovl2* KO mice are male-sterile due to an arrest of spermatogenesis [24], whereas *Elovl5* KO mice exhibit hepatic steatosis [25]. ELOVL3 and ELOVL7 are active towards C16–C22 acyl-CoAs, with the highest activities toward C18–CoAs [9], [26]. Elovl3 is expressed in the skin sebaceous glands and hair follicles, and *Elovl3* KO mice display a sparse hair coat that exhibits a water repulsion defect [27]. Mutations causing inherited diseases have also been found in genes encoding other components of FA elongation machinery. For example, a mutation in the 3-hydroxyacyl-CoA dehydratase *HACD1* gene, which is specifically expressed in skeletal muscle, causes myopathy [28]. In addition, a point mutation in the *trans*-2-enoyl-CoA reductase, *TER*, which causes a Pro182-to-Leu change in the gene product, leads to nonsyndromic mental retardation [29]. This mutation reduces the enzyme activity and stability of the TER protein and causes decreases in sphingolipids with C24 [30].

Despite the increasing evidence for the pathological and physiological importance of FA elongation, knowledge concerning the regulation of FA elongation still remains largely unknown, mainly due to difficulties in biochemical analyses using purified enzymes. All components of the FA elongation machinery are integral membrane proteins with multiple transmembrane domains, which thus must be solubilized with non-ionic detergent before purification. However, this solubilization causes a decrease in enzyme activity of FA elongases [26], and micelles of the non-ionic detergent may prevent possible interactions among components of the FA elongation machinery. Nevertheless, we recently succeeded in the biochemical characterization of purified ELOVL7 by reconstituting the protein into proteoliposomes [26]. In the present study, we expanded such biochemical analysis to ELOVL6 and also examined the effect of including KAR, which is responsible for the reduction step following the condensation step by ELOVLs, in the same proteoliposomes. We found that KAR largely stimulated ELOVL6 activity. Furthermore, this stimulation was divided into either KAR enzyme activity-independent or -dependent mechanisms. With respect to the activity in a KAR enzyme activity-independent manner, the existence of KAR may cause a conformational change in ELOVL6, probably through direct interaction, leading to increased enzyme activity. With respect to the activity in a KAR enzyme activity-dependent manner, KAR may stimulate the removal of products from the active site of ELOVL6 to allow a next-round reaction to occur. Together with our previous findings that the fourth-step enzyme, TER, affects the enzyme activity of the third-step enzyme, HACDs, in the FA elongation cycle [30], these results indicate that components of the FA elongation machinery work in a coordinated manner.

## Materials and Methods

### Cell Culture and Transfection

HEK 293T and HeLa cells were grown in Dulbecco's modified Eagle's medium (D6429 for HEK 293T cells and D6046 for HeLa cells; Sigma, St. Louis, MO) supplemented with 10% fetal bovine serum, 100 units/ml penicillin, and 100 µg/ml streptomycin. HEK 293T cells were grown in dishes coated with 0.3% collagen. Transfections were performed using Lipofectamine Plus Reagent (Life Technologies, Carlsbad, CA) according to the manufacturer's instructions.

### Yeast Strains and Media

The yeast *Saccharomyces cerevisiae* strain used in this study was derived from BY25598 (*MATa his3–11,15 leu2–3,112 ura3::PADH-OsTIR1* (*URA3*) *trp1-1 ade2-1 can1-100*) [31]. TatY42 (*YBR159W-FLAG-AID*) was constructed by chromosomal fusion of a coding sequence for tandemly oriented FLAG and auxin-inducible degron (AID) with the *YBR159W* gene from BY25598 as described previously [32]. The *ayr1*Δ*::natNT2* mutation was then introduced into the TatY42, generating TatY56 (*YBR159W-FLAG-AID ayr1*Δ*::natNT2*). Cells were grown in synthetic complete (SC) medium (0.67% yeast nitrogen base, 2% D-glucose, and nutritional supplements) lacking tryptophan (SC-Trp) at 30°C.

### Plasmids

pCE-puro 3xFLAG-1 is a mammalian expression vector designed to produce an N-terminal 3xFLAG-tagged protein. pCE-puro 3xFLAG-ELOVL6 and pCE-puro 3xFLAG-KAR are derivatives of the pCE-puro 3xFLAG-1 vector and encode human *3xFLAG-ELOVL6* and 3*xFLAG-KAR*, respectively [9]. pWK40 (*CEN*, *TRP1* marker) is a yeast expression vector designed to produce an N-terminal 2xHA-tagged protein under the control of the *GAPDH* promoter. The pTN41 (*2xHA-YBR159w*), pTN42 (*2xHA-KAR*), pTN44 (*2xHA-KAR Y202A/K206A*), pTN45 (*2xHA-KAR S189A*), and pTN46 (*2xHA-KAR N161A*) plasmids were generated by cloning respective genes into the pWK40 vector. The *KAR* mutants were created using a QuikChange site-directed mutagenesis kit (Stratagene, Agilent Technologies, La Jolla, CA) with the following primers: for *KAR Y202A/K206A*,

5′-CTTGACCATCGCTTCTGCAACCGCGACTTTTGTAG-3′ and 5′-CTACAAAAGTCGCGGTTGCAGAAGCGATGGTCAAG-3′; for *KAR S189A*, 5′-GGCTATTCTGAACATTTCAGCTGGCAGTGGCATGC-3′ and 5′-GCATGCCACTGCCAGCTGAAATGTTCAGAATAGCC-3′; and for *KAR N161A*, 5′-GAAAATGATAAATATTGCTATTCTTTCTGTTTG-3′ and 5′-CAAACAGAAAGAATAGCAATATTTATCATTTTC-3′). The pCE-puro 3xFLAG-KAR S189A plasmid was constructed by cloning of the *3xFLAG-KAR S189A* gene from the pTN45 plasmid into the pCE-puro 3xFLAG-1 vector.

### Purification of 3xFLAG-tagged Proteins

HEK 293T cells were transfected with the pCE-puro 3xFLAG-ELOVL6, pCE-puro 3xFLAG-KAR, or pCE-puro 3xFLAG-KAR S189A plasmid. Twenty-four hours after transfection, cells were suspended in buffer A (50 mM HEPES-NaOH (pH 6.8), 500 mM NaCl, 10% glycerol, 1 mM dithiothreitol, 1 mM phenylmethylsulfonyl fluoride, and a 1× protease inhibitor mixture (Complete EDTA-free; Roche Diagnostics, Indianapolis, IN) and lysed by sonication. After removal of cell debris by centrifugation (2,000 × g, 3 min, 4°C), the supernatant was centrifuged at 100,000×g for 30 min at 4°C. The resulting pellet (total membrane fraction) was suspended in buffer A containing 2% Triton X-100, followed by rotation for 30 min at 4°C to stimulate solubilization. After insolubilized proteins were removed by centrifugation (100,000×g, 30 min, 4°C), the supernatant (solubilized fraction) was incubated with anti-FLAG M2 agarose (Sigma) by rotating at 4°C overnight. The beads were washed twice with buffer A containing 2% TritonX-100, once with buffer A containing 1% TritonX-100, once with buffer A containing 0.5% TritonX-100, and twice with buffer A containing 0.2% TritonX-100. The bound proteins were then eluted with buffer A containing 0.2% Triton X-100 and 100 µg/ml 3xFLAG peptide.

### *In vitro* FA Elongation Assay

Reconstitution of proteins into proteoliposomes was performed as follows. For preparation of the liposomal lipid, 1-palmitoyl-2-oleoylphosphatidylcholine (Avanti Polar Lipids, Alabaster, AL) in chloroform was dried, suspended in buffer B (50 mM HEPES-NaOH (pH 6.8), 500 mM NaCl, 10% glycerol, 1 mM dithiothreitol), incubated at 50°C for 1 h, sonicated, treated with 2% Triton X-100, and sonicated again. Purified proteins were diluted with buffer A containing 0.2% TritonX-100 to 100 µl and mixed with 20 µl of the 10 mg/ml phosphatidylcholine solution. After three repeats of 20 times mixing by pipetting and incubation on ice for 15 min, the mixture was incubated with 30 µl Bio-beads SM-2 (Bio-Rad, Hercules, CA) at 4°C for 5 h while mixing to remove Triton X-100.

*In vitro* FA elongation assays were performed as described previously [9], [33]. Acyl-CoA substrates purchased from Sigma or Avanti Polar Lipids were first suspended in water to 10 mM and then diluted to 0.4 mM in buffer A containing 4 mg/ml FA-free bovine serum albumin (Sigma). Proteoliposomes were incubated with 1 µl of 0.4 mM acyl-CoA (final concentration 8 µM) and 0.075 µCi [^14^C]malonyl-CoA (55 mCi/mmol; Moravek Biochemicals, Brea, CA) for 30 min or 90 min at 37°C in a 50-μl reaction mixture (50 mM HEPES-NaOH (pH 6.8), 150 mM NaCl, 10% glycerol, 1 mM dithiothreitol, 2 mM MgCl_2_, 1 mM CaCl_2_, 1 mM phenylmethylsulfonyl fluoride, and a 1× protease inhibitor mixture) in the presence or absence of 1 mM NADPH. After the reaction, the samples were treated with 25 µl of 75% KOH (w/v) and 50 µl of ethanol and incubated at 70°C for 1 h to release CoA. After acidification by adding 100 µl 5 N HCl and 50 µl of ethanol, lipids were extracted with 700 µl of hexane, dried, suspended in 20 µl of chloroform, and separated by normal-phase TLC on Silica Gel 60 TLC plates (Merck, Whitehouse Station, NJ) with hexane/diethyl ether/acetic acid (30∶70∶1, v/v) as the solvent system. Methyl-esterification of FAs and separation by reverse-phase TLC was performed as described previously [9].

### Deglycosylation of Proteins

Endoglycosidase H (Endo H) and peptide: *N*-glycosidase F (PNGase F) were purchased from New England Biolabs (Beverly, MA). Deglycosylation assays were performed according to the manufacturer's instructions.

### Immunoblotting

Immunoblotting was performed as described previously [34], using anti-FLAG antibody M2 (1 µg/ml; Stratagene) as the primary antibody and HRP-conjugated anti-mouse IgG F(ab’)_2_ fragment (1∶7500 dilution; GE Healthcare Life Sciences, Buckinghamshire, UK) as the secondary antibody. Labeling was detected using Pierce Western Blotting Substrate (Thermo Fisher Scientific, Waltham, MA) or Western Lightning Plus-ECL (PerkinElmer Life Science, Waltham, MA).

## Results

### Progression of the FA Elongation Cycle Enhances ELOVL6 Activity

We previously reported that NADPH enhanced the enzyme activity of ELOVL7 *in vitro* using total membrane fractions prepared from HEK 293T cells overproducing ELOVL7 [26]. Although ELOVL7 itself does not require NADPH for its catalytic reaction, the second and fourth reactions in the FA elongation cycle catalyzed by KAR and TER, respectively, need NADPH as a reducing agent (Fig. 1A). This result suggests that progression of the FA elongation cycle somehow enhances ELOVL7 activity. However, the molecular mechanism underlying the enhancement still remains unclear. In addition, it is also unsolved which or both of the reducing reactions are involved in the enhancement.

In the present study, we first examined whether such enhancement was specific to ELOVL7 or was common to other ELOVL-mediated reactions. For this purpose, membrane fractions prepared from HEK 293T cells overproducing 3xFLAG-ELOVL6 were subjected to an *in vitro* FA elongation assay, where the membrane fractions were incubated with the substrates (palmitoyl-CoA and [^14^C]malonyl-CoA) in the presence or absence of NADPH. After the reaction, products were saponified to convert acyl-CoAs to FAs and separated by TLC. In the absence of NADPH, 3-ketoacyl-CoA (3-keto FA and its by-product during saponification, marked by an asterisk in Fig. 1B) was the sole product (Fig. 1B), indicating that only the first step of the FA elongation cycle proceeded. In the presence of NADPH, on the other hand, the major product was acyl-CoA (FA in the figure) (Fig. 1B). A small amount of 3-hydroxyacyl-CoA was also produced, whereas no 3-ketoacyl-CoA or *trans*-2-enoyl-CoA was detected. This result was consistent with a previous report that the first condensation step is rate-limiting [35]. The product levels (acyl-CoA plus 3-hydroxyacyl-CoA) in the presence of NADPH were ∼10-fold higher than those (3-ketoacyl-CoA) in the absence of NADPH (Fig. 1C). The increase in product levels was NADPH concentration-dependent, and the product levels reached a maximum at 125 µM (Fig. 1D). These results suggest that ELOVL6 exerts its maximal activity only when the FA elongation cycle proceeds, as is the case with ELOVL7.

### Biochemical Characterization of Purified ELOVL6

**Figure 2:**
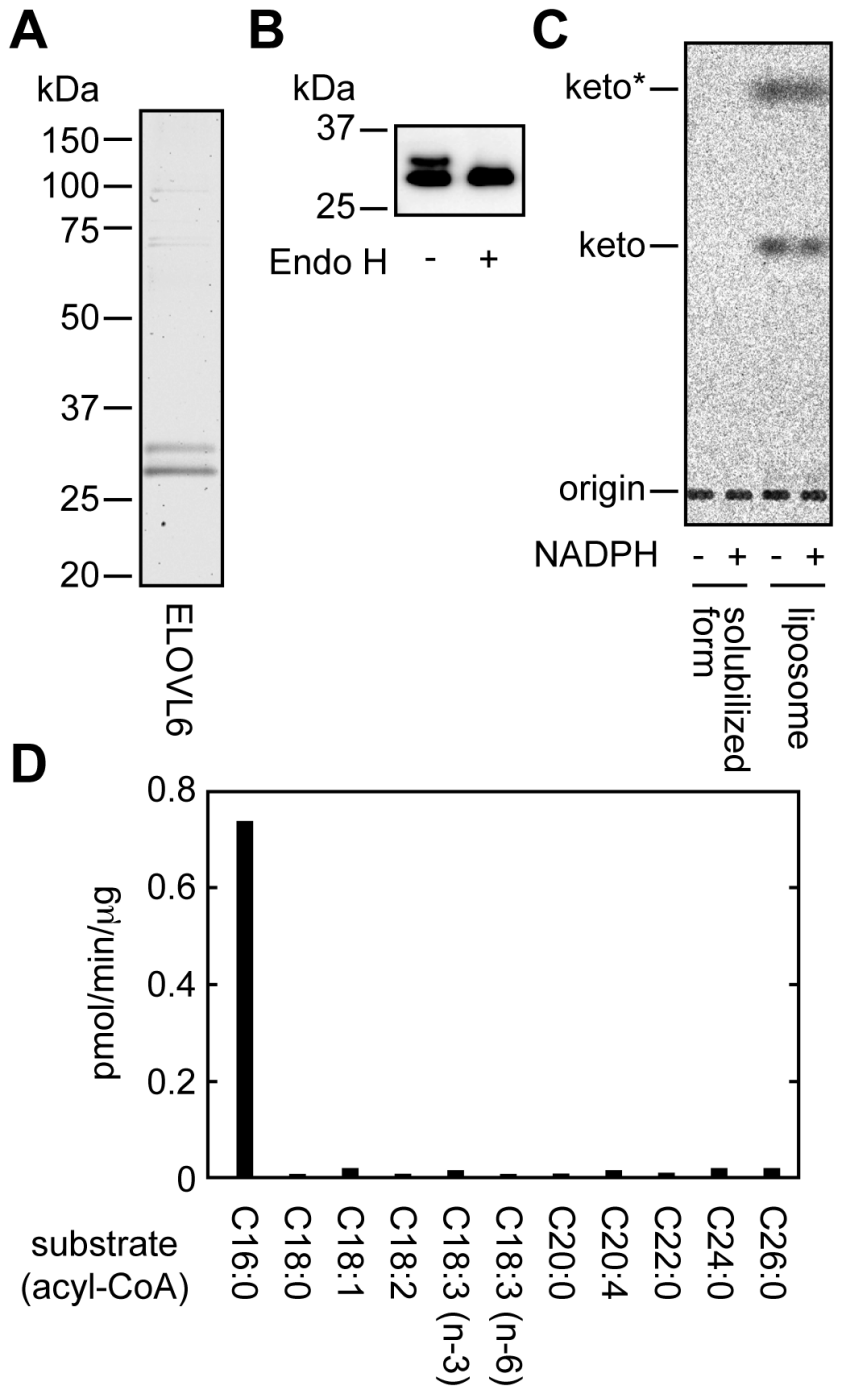
Purified ELOVL6 reconstituted into proteoliposomes exhibits activity toward C16∶0-CoA. (A) Total membrane fractions prepared from HEK 293T cells transfected with the pCE-puro 3xFLAG-ELOVL6 plasmid were solubilized with 2% Triton X-100 and subjected to affinity-purification using anti-FLAG M2 agarose affinity beads. Purified proteins (1 µg) were separated by SDS-PAGE and stained with Coomassie brilliant blue. (B) Purified 3xFLAG-ELOVL6 proteins were incubated with or without Endo H and subjected to immunoblotting with anti-FLAG antibody. (C) Purified 3xFLAG-ELOVL6 proteins (50 ng) in a solubilized state or reconstituted into proteoliposomes were incubated with 8 µM C16∶0-CoA and 27.3 µM [^14^C]malonyl-CoA (1.5 µCi/ml) for 90 min at 37°C in the presence or absence of 1 mM NADPH. After termination of the reactions, lipids were saponified, acidified, extracted, and separated by normal-phase TLC, followed by detection using a bioimaging analyzer BAS-2500. keto, 3-keto-FA; *keto, a by-product of 3-ketoacyl-CoA. (D) Purified 3xFLAG-ELOVL6 proteins (30 ng) reconstituted into proteoliposomes were incubated with the indicated acyl-CoA (8 µM) and 27.3 µM [^14^C]malonyl-CoA (1.5 µCi/ml) for 90 min at 37°C and processed as in (C). Products were detected and quantified with a bioimaging analyzer BAS-2500.

To reveal the mechanism of increased ELOVL6 activity in the presence of NADPH, it was necessary to perform *in vitro* analyses using purified proteins. For this purpose, we first established an *in vitro* assay system using purified 3xFLAG-ELOVL6 protein. 3xFLAG-ELOVL6 was overproduced in HEK 293T cells, solubilized with Triton X-100, and affinity-purified. Coomassie brilliant blue staining detected two bands of 3xFLAG-ELOVL6 at 29 kDa and 32 kDa (Fig. 2A). The upper band represented an *N*-glycosylated form, since it was changed to a non-glycosylated form upon treatment with Endo H (Fig. 2B). Although ELOVL6 had no enzyme activity under solubilized conditions, as is the case with ELOVL7 [26], reconstitution of ELOVL6 into proteoliposomes enabled it to exhibit enzyme activity (Fig. 2C). The enzyme activity of ELOVL6 in the presence of NADPH was similar to that in the absence of NADPH (Fig. 2C), confirming that NADPH is not required for the ELOVL6 activity. We then investigated the substrate specificity of ELOVL6 using 11 different acyl-CoAs. Purified ELOVL6 exhibited specific activity toward C16∶0-CoA (Fig. 2D), consistent with our previous results from using total membrane fractions prepared from HEK 293T cells overexpressing 3xFLAG-ELOVL6 [9].

**Figure 3:**
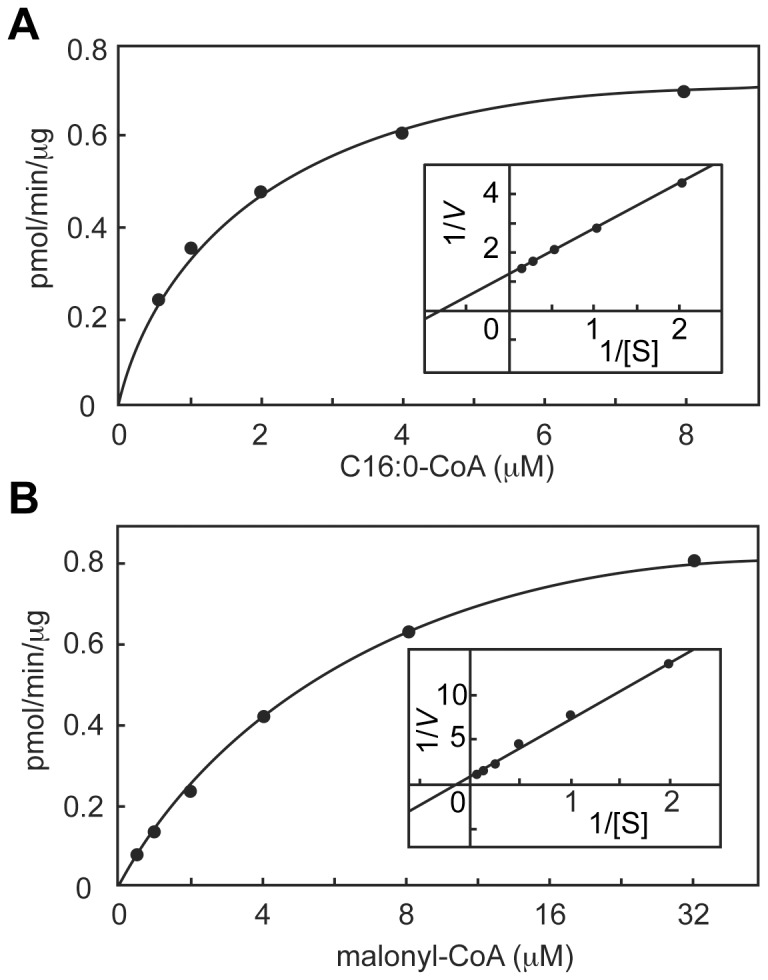
Determination of kinetic parameters of ELOVL6. (A and B) Purified 3xFLAG-ELOVL6 proteins (250 ng) were reconstituted into proteoliposomes and incubated with the indicated concentrations of C16∶0-CoA and 27.3 µM [^14^C]malonyl-CoA (1.5 µCi/ml) (A) or 8 µM C16∶0-CoA and the indicated concentrations of [^14^C]malonyl-CoA (B) for 90 min at 37°C. After termination of the reactions, lipids were saponified, acidified, extracted, and separated by normal-phase TLC, followed by detection and quantification with a bioimaging analyzer BAS-2500. The obtained values were expressed in Michaelis–Menten plots and Lineweaver–Burk plots (insets).

We also determined kinetic parameters of ELOVL6. A FA elongation assay was performed using purified 3xFLAG-ELOVL6 reconstituted into proteoliposomes and various concentrations of C16∶0-CoA. The obtained values were then aligned in a Lineweaver–Burk plot (Fig. 3A). The *K*_m_ and *V*_max_ values were calculated to be 1.22 µM and 0.79 pmol/min/μg protein, respectively. On the other hand, the *K*_m_ and *V*_max_ values for another substrate, malonyl-CoA, were determined to be 6.46 µM and 1.03 pmol/min/μg protein, respectively (Fig. 3B). Comparing our previous results from an examination of the kinetic parameters of ELOVL7 under similar conditions (*K*_m_, 2.6 µM for α-linolenoyl-CoA, which is the best substrate of ELOVL7, and 11.7 µM for malonyl-CoA; *V*_max_, 0.33 pmol/min/μg protein for α-linolenoyl-CoA and 0.31 pmol/min/μg protein for malonyl-CoA) [26], we calculated that the *V*_max_ values of ELOVL6 were 2.4–3.3-fold higher than those of ELOVL7, whereas its *K*_m_ values were ∼2-fold lower. The higher enzyme activity of ELOVL6 as compared to ELOVL7 was consistent with the results of the *in vitro* FA elongation assay using membrane fractions [9]. From these results, we concluded that our purified system operated adequately.

### A 3-Ketoacyl-CoA Reductase Enhances the Enzyme Activity of ELOVL6

**Figure 4:**
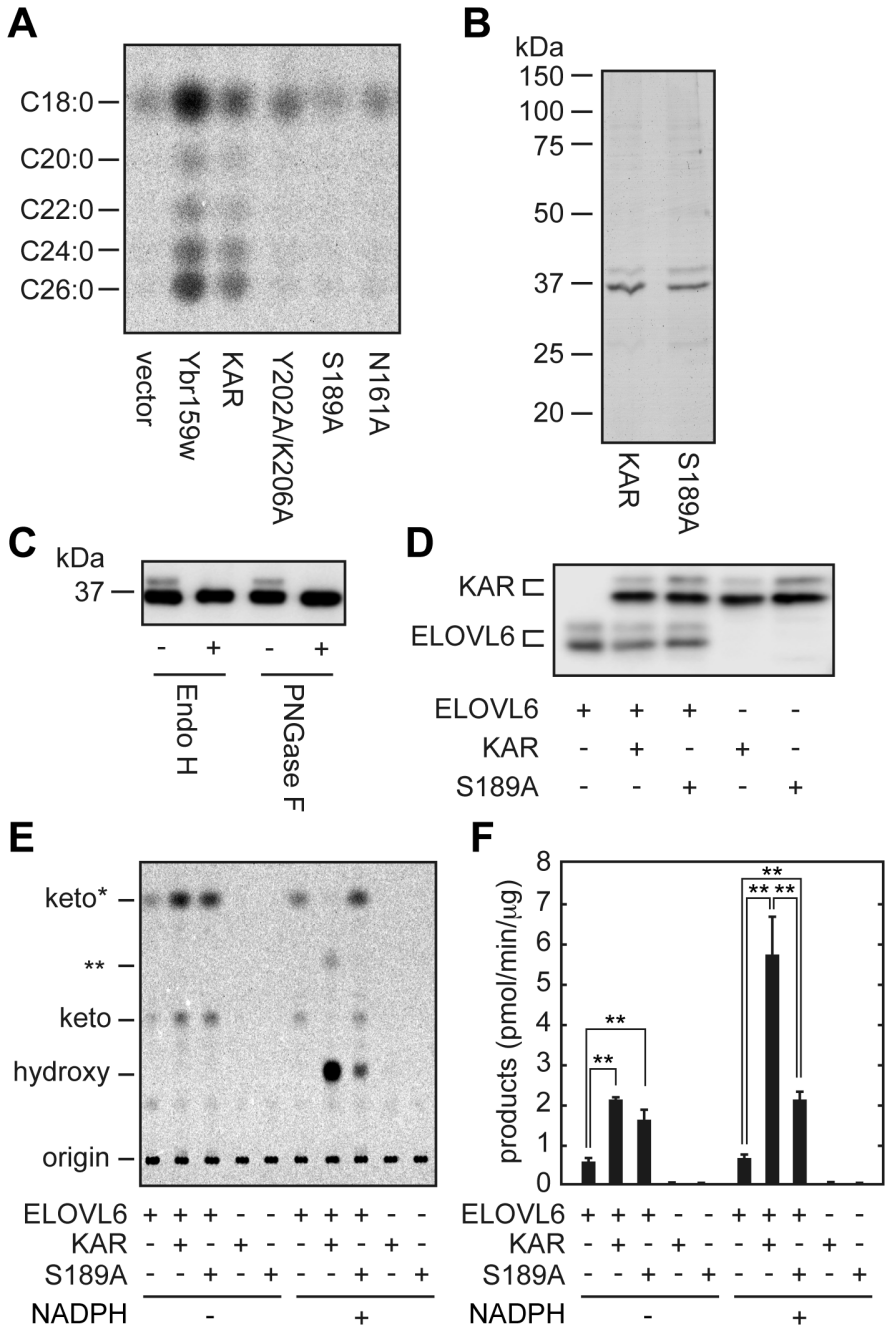
KAR stimulates ELOVL6 enzyme activity in KAR enzyme activity-independent and -dependent manners. (A) The yeast TatY56 (*ayr1*Δ*::natNT2 YBR159W-FLAG-AID*) cells harboring the pWK40 (2xHA vector), pTN41(*2xHA-YBR159w*), pTN42 (*2xHA-KAR*), pTN44 (*2xHA-KAR Y202A/K206A*), pTN45 (*2xHA-KAR S189A*), or pTN46 (*2xHA-KAR N161A*) plasmid were treated with 0.5 mM 3-indolacetic acid for 2 h. Total membrane proteins (20 µg) prepared from them were incubated with 20 µM C16∶0-CoA, 73 µM malonyl-CoA, and 27 µM [^14^C]malonyl-CoA (0.075 µCi) for 30 min at 37°C in the presence of 1 mM NADPH. After termination of the reactions, lipids were saponified, acidified, extracted, converted to methyl ester forms, separated by reverse-phase TLC, and detected by a bioimaging analyzer BAS-2500. (B) Total membrane fractions prepared from HEK 293T cells transfected with the pCE-puro 3xFLAG-KAR or pCE-puro 3xFLAG-KAR S189A plasmid were solubilized with 2% Triton X-100, and subjected to affinity-purification using anti-FLAG M2 agarose affinity beads. Purified proteins (1 µg) were separated by SDS-PAGE and stained with Coomassie brilliant blue. (C) Purified 3xFLAG-KAR proteins (15 ng) were treated with Endo H or PNGase F and subjected to immunoblotting using an anti-FLAG M2 antibody. (D-F) Purified 3xFLAG-ELOVL6 (10 ng) and/or 3xFLAG-KAR proteins of either wild type or S189A mutant (20 ng) were reconstituted into proteoliposomes and subjected to immunoblotting using an anti-FLAG M2 antibody (D) or to an *in vitro* FA elongation assay (E and F). (E) Proteoliposomes were incubated with 8 µM C16∶0-CoA and 27.3 µM [^14^C]malonyl-CoA (1.5 µCi/ml) for 90 min at 37°C in the presence or absence of 1 mM NADPH. After termination of the reactions, lipids were saponified, acidified, extracted, and separated by normal-phase TLC, followed by detection using a bioimaging analyzer BAS-2500. keto, 3-keto-FA; *keto, a by-product of 3-ketoacyl-CoA; hydroxy, 3-hydroxy-FA; **, *trans*-2-enoyl-FA produced through non-enzymatic conversion or produced by contaminated 3-hydroxyacyl-CoA dehydratase. (F) Values presented are the sum of the reaction products in (E) and represent the mean ± S.D. from three independent experiments. Statistically significant differences are indicated (**p<0.01; *t*-test).

Using the assay system established as described above, we next examined the involvement of KAR in the stimulation of ELOVL6 enzyme activity. KAR belongs to the short-chain dehydrogenase/reductase (SDR) superfamily [36], whose members commonly contain catalytic tetrads consisting of Asn, Ser, Tyr, and Lys residues [37]. In KAR, the Asn161, Ser189, Tyr202, and Lys206 residues correspond to this tetrad. We created single (N161A and S189A) and double (Y202A/K206A) Ala-substituted KAR mutants and examined their activities using a yeast expression system. FA elongation machinery is conserved between human and yeast, and yeast contains the same enzyme sets as in humans (FA elongases Elo2/Fen1 and Elo3/Sur4; 3-ketoacyl-CoA reductases, Ybr159w and Ayr1; 3-hydroxyacyl-CoA dehydratase, Phs1; and *trans*-2-enoyl-CoA reductase, Tsc13). We previously demonstrated that human enzymes belonging to the FA elongation machinery could substitute for several yeast enzymes (ELOVL1, 3, and 7 for Elo2 and Elo3; HACD1 and 2 for Phs1; and TER for Tsc13) [9], [11], [30]. Of the two yeast 3-ketoacyl-CoA reductases, Ybr159w is the major isozyme. Deletion of the *YBR159w* gene alone causes severe growth defects; double deletion of *YBR159w* and *AYR1* is lethal [38]. Therefore, we utilized the auxin-inducible degron (AID) system [31] to generate a conditional yeast *YBR159w* mutant, in which the Ybr159w-AID protein is transiently degraded upon treatment with auxin 3-indolacetic acid. The *YBR159w-AID ayr1*Δ cells harboring a vector or the plasmid encoding yeast *YBR159w*, wild type *KAR*, or each of the mutant *KAR* genes were subjected to an *in vitro* FA elongation assay using C16∶0-CoA and [^14^C]malonyl-CoA in the presence of NADPH. The *YBR159w-AID ayr1*Δ cells bearing a vector produced only a small amount of C18∶0-CoA, whereas the introduction of yeast *YBR159w* or human *KAR* resulted in increases in C18∶0-CoA levels and generations of longer acyl-CoAs up to C26 (Fig. 4A). On the other hand, all of the created KAR mutants (N161A, S189A, and Y202A/K206A) exhibited little activity. Among the mutants, the S189A mutant exhibited the lowest activity, and produced C18∶0-CoA levels of S189A mutant-expressing cells were almost indistinguishable from those of vector-transfected cells. Thus, the presumed catalytic tetrad residues of KAR all appear important for the enzyme activity.

We purified wild type and S189A mutant KAR, each tagged with 3xFLAG at the N-terminus. In addition to the bands with expected molecular mass of 3xFLAG-KAR (36 kDa), other faint bands were detected at 39 kDa for both wild type and S189A mutant KAR by Coomassie brilliant blue staining (Fig. 4B). These upper bands represented *N*-glycosylated KAR proteins, since they disappeared upon treatment with Endo H or PNGase F (Fig. 4C).

Purified 3xFLAG-ELOVL6 protein and/or 3xFLAG-KAR protein of either wild type or S189A mutant were then reconstituted into proteoliposomes (Fig. 4D) and subjected to a FA elongation assay. Proteoliposomes containing ELOVL6 alone produced only 3-ketoacyl-CoA in both the presence and absence of NADPH at similar levels (Fig. 4E and F). Co-reconstitution of ELOVL6 with KAR into proteoliposomes resulted in a ∼3-fold increase in the 3-ketoacyl-CoA levels compared to ELOVL6 alone in the absence of NADPH. Furthermore, inclusion of wild type KAR with ELOVL6 in the same proteoliposomes caused production of 3-hydroxyacyl-CoA with a large increase in product levels (∼8-fold increase) in the presence of NADPH. The stimulation effect of KAR S189A was similar to that of wild type KAR in the absence of NADPH (Fig. 4E and F). In the presence of NADPH, ELOVL6 and KAR S189A produced only a slight amount of 3-hydroxyacyl-CoA due to the low enzyme activity of KAR S189A (Fig. 4A), and the total product levels were similar to those in the absence of NADPH (Fig. 4E and F). These results indicate that stimulatory effects of KAR on ELOVL6 can be divided into two manners: enzyme activity-dependent and -independent.

**Figure 5:**
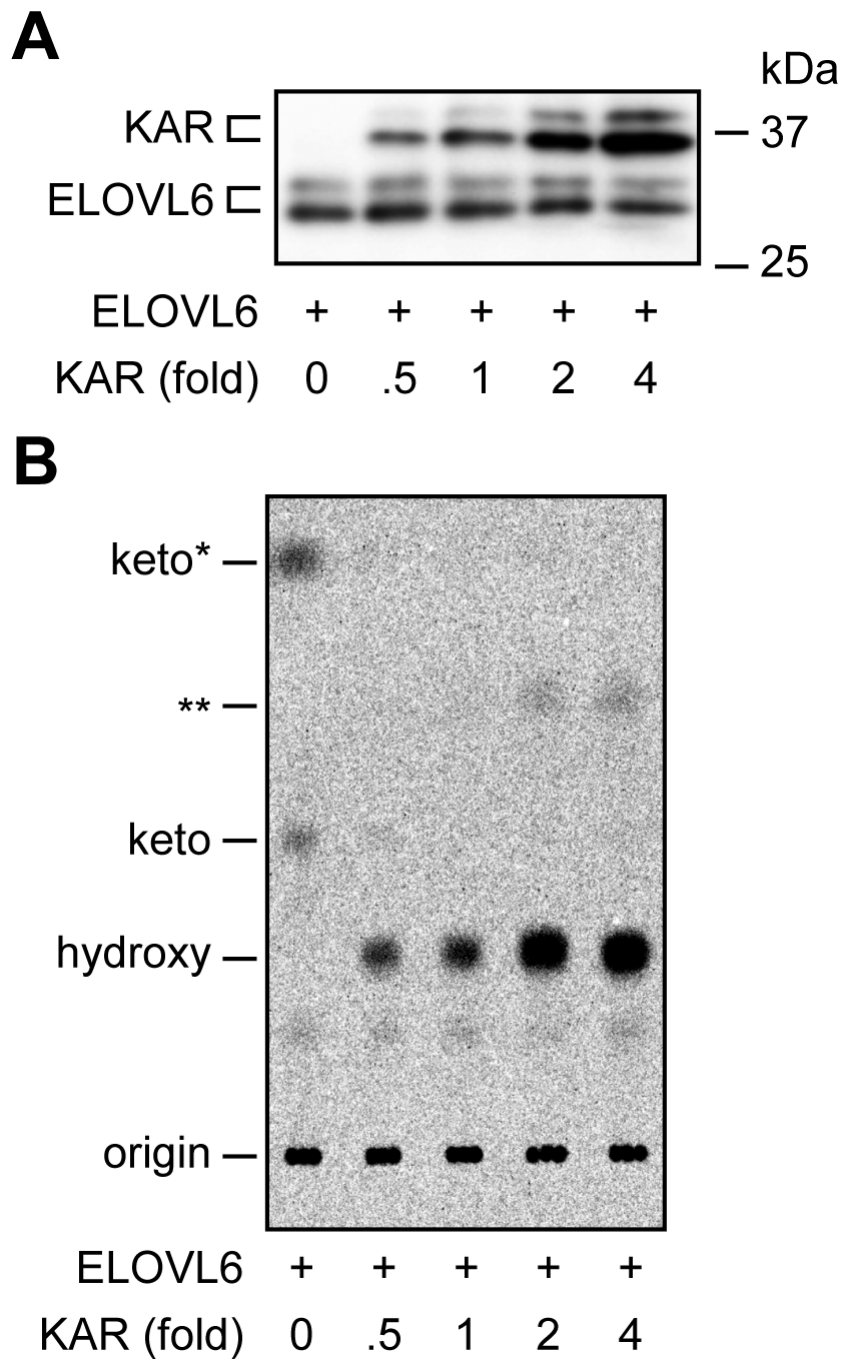
ELOVL6 activity is increased, depending on the amount of KAR, but in a saturable manner. (A and B) Purified 3xFLAG-ELOVL6 (10 ng) and various amounts of 3xFLAG-KAR (0, 5, 10, 20, 40 ng) proteins were reconstituted into proteoliposomes and subjected to immunoblotting using an anti-FLAG M2 antibody (A) or to an *in vitro* FA elongation assay (B). An *in vitro* FA elongation assay was performed by incubating proteoliposomes with 8 µM C16∶0-CoA and 27.3 µM [^14^C]malonyl-CoA (1.5 µCi/ml) for 90 min at 37°C in the presence of 1 mM NADPH. After termination of the reactions, lipids were saponified, acidified, extracted, and separated by normal-phase TLC, followed by detection using a bioimaging analyzer BAS-2500.

We next prepared proteoliposomes, in which ELOVL6 and various amounts of wild type KAR (×0.5, ×1, ×2, and ×4 amounts compared with ELOVL6 levels) were reconstituted (Fig. 5A). The product levels were increased, depending on the amounts of KAR, but they reached a plateau at the ×2 KAR (Fig. 5B). These results suggest that almost all ELOVL6 enzymes complexed with KAR when there was ×2 KAR compared with ELOVL6 levels, so no further KAR could yield any effects.

## Discussion

Although almost all FAS products are a single molecule (palmitic acid; C16∶0) [39], a series of elongation and desaturation cycles create plenty of additional FA species, whose diversity is quite physiologically and pathologically important [2]. Since the FA elongation cycle consists of four distinct enzyme reactions, these reactions must be tuned mutually to drive the cycle efficiently. However, the molecular mechanism regulating FA elongation still remains unclear. In the present study, we focused on the regulation of one of the FA elongases, ELOVL6. ELOVL6 is a key enzyme to produce C18 FAs [9], [12], [13], which are abundant FA species and account for roughly half of mammalian FA levels [1].

We had previously reported that activity of another FA elongase, ELOVL7, was enhanced in the presence of NADPH using membrane fractions, although ELOVL7 itself did not need NADPH for its enzyme reaction [26]. This stimulatory effect of NADPH appears to be common to ELOVLs, since a similar effect was also observed in the present study for ELOVL6 (Fig. 1B and C). NAPDH is a cofactor for the second and fourth reactions of the FA elongation cycle, which are catalyzed by the 3-ketoacyl-CoA reductase KAR and the *trans*-2-enoyl-CoA reductase TER, respectively. To date, it has yet to be determined if progression of one of these reactions or both reactions is responsible for the stimulation of ELOVL activities. In the present study, we reproduced NADPH-dependent stimulation of ELOVL6 activity in a purified system using only ELOVL6 and KAR (Fig. 4E and F). Thus, our results suggest that KAR is involved in the enhancement of ELOVL activities.

The stimulatory effect was observed in both KAR enzyme activity-independent and -dependent contexts. ELOVL6 activity was enhanced by ∼3-fold by the presence of enzymatically inactive states of KAR: wild type KAR in the absence of NADPH and KAR S189A mutant both in the presence and absence of NADPH also displayed enhanced activity (Fig. 4E and F). We speculate that the presumed ELOVL6–KAR interaction causes conformational change in ELOVL6, leading to formation of a proper active site. To date, technical limitations have meant there has been no definitive evidence of interactions between ELOVLs and KAR. Some ELOVLs have been shown to be co-immunoprecipitated with KAR using overproducing proteins [9], [40]. However, since both ELOVLs and KAR are integral membrane proteins with multiple transmembrane domains, the possibility of nonspecific hydrophobic interaction has not been excluded. Furthermore, since that experiment was performed under Triton X-100-solubilized conditions, it was possible that co-immunoprecipitation was detected within the same micelle simply due to coincidence, not due to direct interaction. However, the presumed ELOVL–KAR interaction is highly likely, based on circumstantial evidence of enhancement of ELOVL6 activity by inclusion of KAR in the same proteoliposomes that was observed in this study.

With respect to the enzyme activity in a KAR enzyme activity-dependent manner, ELOVL6 activity was fully stimulated (Fig. 4E and F). This effect was observed only for the case of co-reconstitution of ELOVL6 with wild type KAR under the assay conditions including NADPH. The stimulatory effect was dependent on the KAR amount but was saturated at KAR levels two times ELOVL6 levels (Fig. 5B). This saturation also supports the existence of an ELOVL–KAR complex, since no further enhancement would be expected when all ELOVL proteins have already interacted with KAR. Although the exact molecular mechanism of the enzyme activity in the KAR enzyme activity-dependent enhancement of ELOVL6 activity is still unclear, we hypothesize that conversion of 3-ketoacyl-CoA to 3-hydroxyacyl-CoA by KAR stimulates release of the product from the presumed ELOVL–KAR complex. Without this conversion, 3-ketoacyl-CoA may be stuck within ELOVL6 or the KAR active site and prevent the next round of reactions. In the *in vitro* FA elongation assay using membrane fractions in the presence of NADPH, we detected major acyl-CoA products and minor 3-hydroxyacyl-CoA intermediates, but not 3-ketoacyl-CoA or *trans*-2-enoyl-CoA intermediates (Fig. 1B). This result suggests that conversions of 3-ketoacyl-CoA to 3-hydroxyacyl-CoA and of *trans*-2-enoyl-CoA to acyl-CoA are quite rapid, which may be achieved by direct product-substrate transfers between ELOVL and KAR as well as between HACD and TER. On the other hand, detection of the 3-hydroxyacyl-CoA intermediate suggests that conversion of 3-hydroxyacyl-CoA to *trans*-2-enoyl-CoA is rather slow, or even that it is possible that 3-hydroxyacyl-CoA is released into the lipid bilayer, not directly transferred to HACDs. Although KAR or TER is a single enzyme, four HACD isozymes exist (HACD1–4) [11]. Therefore, release of 3-hydroxyacyl-CoA into the lipid bilayer might enable 3-hydroxyacyl-CoA to enter into the appropriate HACD–TER complex. Similar to the ELOVL–KAR interaction, the HACD–TER interaction has not been definitively verified, although co-immunoprecipitation has been reported [41]. However, circumstantial evidence again suggests that complex formation between HACD and TER is highly likely. We recently reported that membrane fractions prepared from B-lymphoblastoid cells with a *TER* mutation, which affects enzyme activity and stability of the TER protein, or those prepared from HeLa cells treated with *TER* siRNA, exhibited lower HACD activities compared to their respective control cells [30]. These results suggest that HACDs that cannot be complexed with TER are unstable or inactive. From these results we speculate that the FA elongation machinery contains two subcomplexes: ELOVL–KAR and HACD–TER.

Our findings here provide a clue to understanding the molecular mechanisms of how the protein components of the FA elongation machinery concertedly work. Such concerted actions must proceed with four distinct enzyme processes efficiently. Similar harmonized actions are observed for FAS, which catalyzes essentially an identical four-step cycle. Mammalian FAS is a multifunctional enzyme with seven distinct domains, so all of the catalytic domains responsible for FA elongation are located in a single polypeptide [39], [42]. Coincidence of four domains enables the reactions to proceed without releasing the intermediates. In contrast to soluble FAS, all components of the FA elongation machinery are integral membrane proteins. Therefore, structural analyses are difficult to perform with present techniques. Future studies are needed to examine the overall regulation of the FA elongation machinery and the mutual regulation between the putative ELOVL–KAR and HACD–TER subcomplexes.
